# Using cryo-EM to understand antimycobacterial resistance in the catalase-peroxidase (KatG) from *Mycobacterium tuberculosis*

**DOI:** 10.1016/j.str.2020.12.008

**Published:** 2021-08-05

**Authors:** Asma Munir, Michael T. Wilson, Steven W. Hardwick, Dimitri Y. Chirgadze, Jonathan A.R. Worrall, Tom L. Blundell, Amanda K. Chaplin

**Affiliations:** 1 Department of Biochemistry, University of Cambridge, Cambridge, CB2 1GA, UK; 2 CryoEM Facility, Department of Biochemistry, University of Cambridge, Cambridge, CB2 1GA, UK; 3 School of Life Sciences, University of Essex, Wivenhoe Park, Colchester CO4 3SQ, UK

**Keywords:** catalase, peroxidase, heme, cryo-EM, disorder, drug binding, *Mycobacterium tuberculosis*, hydrogen-peroxide, resistance mutations

## Abstract

Resolution advances in cryoelectron microscopy (cryo-EM) now offer the possibility to visualize structural effects of naturally occurring resistance mutations in proteins and also of understanding the binding mechanisms of small drug molecules. In *Mycobacterium tuberculosis* the multifunctional heme enzyme KatG is indispensable for activation of isoniazid (INH), a first-line pro-drug for treatment of tuberculosis. We present a cryo-EM methodology for structural and functional characterization of KatG and INH resistance variants. The cryo-EM structure of the 161 kDa KatG dimer in the presence of INH is reported to 2.7 Å resolution allowing the observation of potential INH binding sites. In addition, cryo-EM structures of two INH resistance variants, identified from clinical isolates, W107R and T275P, are reported. In combination with electronic absorbance spectroscopy our cryo-EM approach reveals how these resistance variants cause disorder in the heme environment preventing heme uptake and retention, providing insight into INH resistance.

## Highlights

• A cryo-EM structure to 2.7 Å resolution of *M. tuberculosis* KatG with isoniazid
• Cryo-EM is able to visualize multiple dynamic binding modes of isoniazid to KatG
• Structural disorder in isoniazid resistance mutations is observed
• Structural disorder of the resistance mutations results in the lack of heme retention

## Graphical abstract

**Figure:**
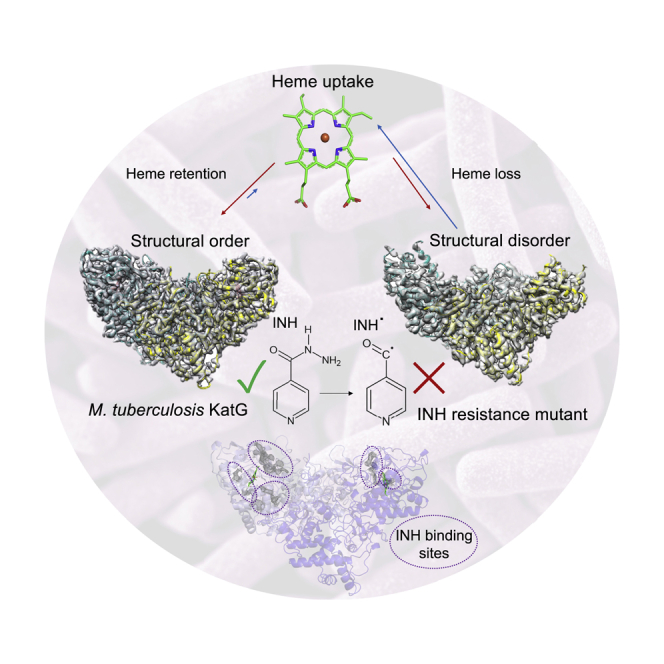

## Teaser

KatG from *Mycobacterium tuberculosis* is a catalase-peroxidase responsible for the activation of the pro-drug isoniazid (INH). Using cryo-EM, Munir et al. demonstrate that binding sites for INH can be visualized. Furthermore, structural disorders in KatG INH resistance mutations are detected using cryo-EM and are linked to heme retention.

## Introduction

Within the past decade the cryoelectron microscopy (cryo-EM) “Resolution Revolution” has dramatically advanced the field of structural biology (Kuhlbrandt, 2014). Cryo-EM technology is continually evolving, with improvements in microscope optics, software for data analysis, sample preparation, and electron detectors, high-resolution structures are being determined routinely. These advances are making it possible to solve the structures of large complex macromolecular assemblies, as well as proteins smaller than 100 kDa and to visualize proteins within living cells using cryoelectron tomography (Rivera-Calzada and Carroni, 2019). Cryo-EM is also increasingly used as a technique to visualize drug binding events within proteins, a research area that was traditionally conducted using X-ray crystallography (Ceska et al., 2019; Merk et al., 2016). Furthermore, cryo-EM advances also now make it possible to visualize a single amino acid substitution within a protein structure, which is of tremendous value when investigating point mutations implicated in drug resistance (Kim et al., 2019).

**Figure 1:**
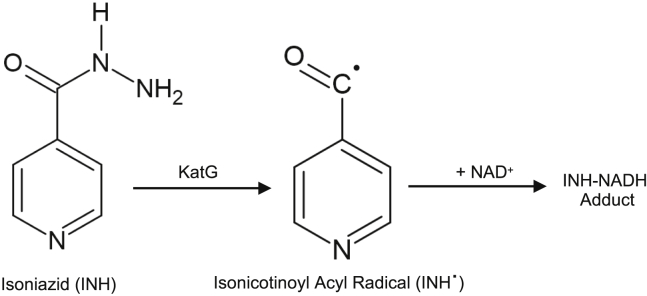
Chemical structure of INH INH is activated in the presence of peroxide by KatG to form an isonictinoyl acyl radical followed by reaction with NAD+ to form the INH-NADH adduct.

In this study we utilize the outlined advances in cryo-EM to study both resistance mutations and drug binding in the KatG protein from *Mycobacterium tuberculosis*, which has been implicated in drug resistance within this pathogen. *M*. *tuberculosis* is the causative agent of TB, responsible for ∼1.2 million deaths every year (Global tuberculosis report, 2020). The public health crisis surrounding TB is exacerbated by a growing number of cases of multi-drug-resistant, extensively drug-resistant, and isonicotinic acid hydrazide (INH)-resistant TB. INH (Figure 1) is a pro-drug, with a molecular weight of ∼137 Da, that has been used as a first-line treatment for TB for over 60 years (Jagielski et al., 2014; Torres et al., 2015a, 2015b; Hameed et al., 2020; Kidenya et al., 2018). It is well established that INH requires activation by KatG (Figure 1), a heme-dependent catalase-peroxidase enzyme that can utilize and degrade hydrogen peroxide (H_2_O_2_) either through functioning as a catalase (Equation 1) or as a peroxidase (Equation 2).

(Equation 1) $$2{\text{H}}_{2}{\text{O}}_{2}\to 2{\text{H}}_{2}\text{O}+{\text{O}}_{2}$$

(Equation 2) $${\text{H}}_{2}{\text{O}}_{2}+2\text{INH}\to 2{\text{INH}}^{\cdot }+2{\text{H}}_{2}\text{O}$$

In Equation 2, two molecules of INH can be sequentially oxidized in single-electron processes to form the active INH⋅ (Figure 1).

The enzymatic activity of KatG has been well studied over the past five decades; however, details of the INH activation mechanism are still not fully resolved (Dunford, 2010). It is known, however, that the activated drug species forms an INH-NAD adduct that subsequently triggers anti-tubercular activity. More than 60% of all known INH resistance mutations are found within the gene that encodes KatG (Hazbon et al., 2006; Muthaiah et al., 2017; Zhang et al., 2005; Cardoso et al., 2004). These mutations render the enzyme unable to activate the pro-drug, thus leading to INH resistance. Due to the consistent rise in the prevalence and severity of resistant TB strains, an increased understanding of the relationship between INH-conferring mutations in KatG and their consequences for the structure and mechanism of INH activation is essential.

Extensive studies have previously been carried out on KatG to understand the structure and mechanism of this important enzyme. The X-ray crystal structure of wild-type (WT) KatG was reported in 2004 (Bertrand et al., 2004). The homodimeric structure is predominantly α-helical with each protomer (∼80 kDa) composed of two domains. The core structure is common to many bacterial and plant peroxidase families, including cytochrome *c* peroxidase (C*c*p) (Wang et al., 1990), ascorbate peroxidase (APX) (Sharp et al., 2003), and horseradish peroxidase C (HRPC) (Gajhede et al., 1997). The N-terminal domain contains a heme *b* and substrate binding site, which are essential for enzymatic function. The C-terminal domain is homologous to the N-terminal domain and is necessary for the overall enzyme activity but does not contain a functional heme binding site. The specific role of the C-terminal domain in the function of KatG is not clearly understood (Heym et al., 1993). The heme site is surrounded by six conserved residues: Arg104, Trp107, and His108 in a pocket distal to the heme, and His270, Trp321, and Asp381 in a pocket proximal to the heme. A covalently linked “MYW catalytic triad” is formed by the conserved residues, Met255, Tyr229, and Trp107, which is essential for catalase activity (Jakopitsch et al., 2003, 2004; Santoni et al., 2004).

To date, more than 300 INH resistance mutations have been reported in KatG (Vilcheze and Jacobs, 2014). Enzymatic and spectroscopic studies have shown that many of these mutations affect the oxidation of INH and/or the catalase and peroxidase activity (Cade et al., 2010). However, limited structural information is available for many of these variants, and thus a detailed molecular understanding of how these mutations modulate INH activation and effect catalase and peroxidase activity remains poorly understood. To remedy this gap in information we have sought to assess whether a cryo-EM approach is applicable to studying INH binding and resistance mutations in KatG. Here, we report the cryo-EM structures of WT KatG from *M*. *tuberculosis* with and without INH bound at 2.7 and 3.7 Å resolution, respectively, and have identified an ensemble of potential INH binding sites. We note that no X-ray crystal structure of *M*. *tuberculosis* KatG bound to INH has been reported. Furthermore, we reveal that, for two INH resistance variants, W107R and T275P (Gagneux et al., 2006; Heym et al., 1995), significant structural disorder relating to heme uptake and retention is the likely cause for INH resistance. Cryo-EM therefore provides valuable molecular insight into the dynamics of heme binding that has eluded crystallographic approaches in pursuing molecular insights into resistance variants. This methodology should be broadly applicable for all other KatG variants and could be utilized to study functionally related proteins.

## Results

### Cryo-EM grid optimization

**Figure 2:**
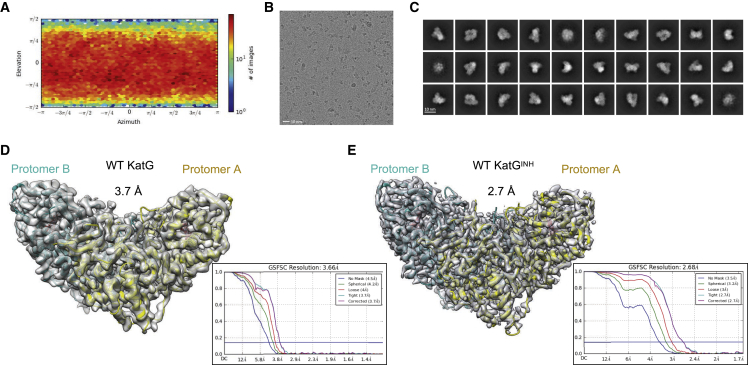
Cryo-EM sample optimization and structures of WT KatG from *M*. *tuberculosis* (A) Angular distribution calculated in cryoSPARC for particle projections following the addition of 3-[(3-cholamidopropyl)dimethylammonio]-1-propanesulfonate, with the heatmap showing the number of particles for each viewing angle. (B) Example of a micrograph of WT KatG. Scale bar, 10 nm. (C) Example of 2D classes of WT KatG. Scale bar, 10 nm. (D) WT homodimeric KatG structure with FSC curves shown as an inset. (E) WT homodimeric KatG^INH^ structure with FSC curves shown as an inset. Cryo-EM maps are shown as a gray semi-transparent surface, protomer A is shown in yellow, and protomer B in cyan.

We first sought to establish whether moderate- to high-resolution structures of KatG could be routinely produced using single-particle cryo-EM. Initial grid preparation efforts showed some promising single particles; however, these grids were also prone to clusters of particle aggregation and preferred orientation problems, with KatG sampling relatively few orientations within the ice. After extensive optimization efforts we found that cryo-EM grids of WT KatG could be enhanced by the addition of 3-[(3-cholamidopropyl)dimethylammonio]-1-propanesulfonate (CHAPSO), which both reduced particle aggregation and eliminated preferred orientation bias (Figures 2A–2C) (Chen et al., 2019). After optimization of the grid preparation method we collected a dataset yielding a cryo-EM structure of WT KatG to 3.7 Å resolution (Figure 2D). However, on addition of the pro-drug INH to WT KatG samples, better quality grids resulting in higher-resolution cryo-EM maps were consistently obtained (2.7 Å, Figure 2E; Table 1). Local resolution maps of the two WT cryo-EM datasets can be seen in Figure S1. Models of KatG with and without INH were constructed using the previously determined X-ray crystallography structure as a starting model (PDB: 2CCA; Zhao et al., 2006). Superposition of the cryo-EM models with and without INH showed no significant structural deviations and, thus, based on the higher resolution of KatG with INH bound (from here on referred to as KatG^INH^), we focus on this model for further detailed analysis.

**Table 1:** Cryo-EM data collection and refinement statistics

	WT EMD-11234 PDB: 6ZJI	WT + 30 mM INH EMD-11776 PDB: 7AG8	W107R as purified (1 heme) EMD-11625 PDB: 7A2I	W107R after heme uptake (1 heme) EMD-11677 PDB: 7A7C	W107R after heme uptake (2 hemes) EMD-11676 PDB: 7A7A	T275P as purified EMD-11680 PDB: 7A8Z	T275P after heme uptake EMD-11689 PDB: 7AA3
**Data collection and processing**							

Detector	Gatan K2	Falcon III	Falcon III	Gatan K3	Gatan K3	Gatan K2	Gatan K2
Magnification	215k	96k	92k	130k	130k	130k	130k
Energy filter slit width (eV)	20	20	20	20	20	20	20
Voltage (kV)	300	300	300	300	300	300	300
Flux on detector (e/pix/s)	4.54	0.56	0.81	14.90	14.90	5.85	5.06
Electron exposure on sample (e−/Å^2^)	50.29	48.60	51.30	39.30	39.30	49.40	50.60
Target defocus range (μm)	1.5–2.4	0.7–2.1	0.7–2.1	0.8–1.6	0.8–1.6	0.8–2.6	1.3–2.8
Calibrated pixel size (Å)	0.64	0.83	0.83	0.652	0.652	1.05	1.05
Symmetry imposed	C1	C1	C1	C1	C1	C2	C2
Extraction box size (pixels)	360	290	270	380	380	280	290
Initial particle images (no.)	182,834	142,649	76,067	244,867	244,867	341,958	211,033
Final particle images (no.)	96,044	89,703	50,043	60,953	71,350	197,074	165,609

**Refinement**							

Map resolution at FSC = 0.143 (Å)^∗^	3.66	2.68	3.30	3.16	3.08	3.35	3.56
Model composition							
Non-hydrogen atoms	10,791	10,920	9,476	9,391	10,150	8,405	8,394
Protein residues	1,397	1,417	1,217	1,226	1,324	1,103	1,094
B factor (Å^2^)							
Protein	144.86	80.74	95.26	67.58	58.60	93.36	75.57
Ligand (heme)	114.89	70.76	95.03	67.09	63.95	–	–
RMSD							
Bond lengths (Å)	0.009	0.008	0.004	0.003	0.009	0.004	0.004
Bond angles (°)	0.813	0.699	0.609	0.523	0.744	0.575	0.647
Validation							
MolProbity score	2.25	1.75	1.84	1.68	1.99	1.86	2.05
Clashscore	12.85	6.61	7.20	6.62	8.88	6.97	9.36
Poor rotamers (%)	0.28	0.09	0.00	0.00	0.00	0.00	0.00
Ramachandran plot (%)							
Favored	86.50	94.26	93.15	95.54	91.02	92.34	89.85
Allowed	13.43	5.67	6.85	4.46	8.98	7.57	10.15
Disallowed	0.07	0.07	0.00	0.00	0.00	0.09	0.00

W107R values after heme uptake are from the same data collection. ^∗^Cryo-EM maps were used to guide model building, final refinement used the gold standard Fourier shell correlation (FSC) = 0.143 maps. All refinement statistics from MolProbity, PHENIX version 1.18.2. RMSD, root-mean-square deviation.

### Cryo-EM structure of KatG^INH^

**Figure 3:**
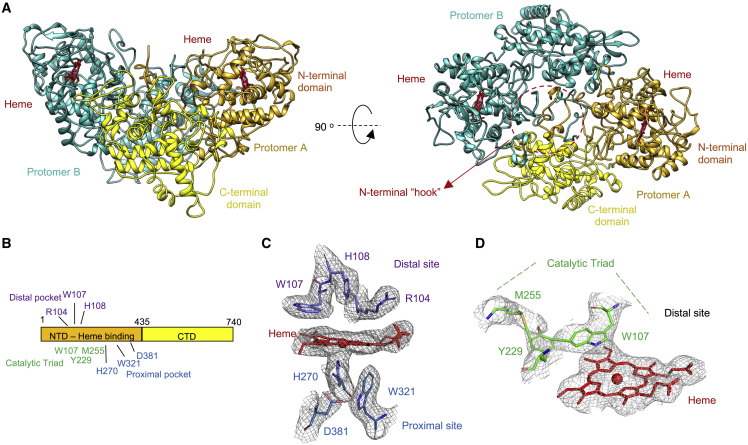
Cryo-EM structure of WT KatG^INH^ (A) Cryo-EM model of KatG^INH^ in two orientations. Protomer A is shown in yellow with the N-terminal domain (NTD) a darker shade than the C-terminal domain (CTD). Protomer B is shown in cyan and heme in red. The N-terminal hook is highlighted in the dashed red circle. (B) Linear schematic of WT KatG with the NTD and CTD colored according to protomer A in (A). Conserved distal and proximal site residues are indicated, and the covalent catalytic triad labeled. (C) The heme environment with distal and proximal heme site residues shown as purple sticks and heme in red. (D) The catalytic triad formed by the MYW (Met255-Tyr229-Trp107) residues. Cryo-EM maps are shown as gray mesh.

The dimeric cryo-EM structure of KatG^INH^ is shown in Figure 3. Stabilization of the homodimer has been suggested to involve the N-terminal residues of each protomer to create a “hook”-like structure, a feature seen in our KatG^INH^ structure (Bertrand et al., 2004) (Figure 3A). During refinement of the KatG^INH^ dimer no symmetry was imposed; however, despite this, it is apparent that the two protomers are nearly identical, with a root-mean-square deviation (RMSD) value of 0.4 Å for Cα atoms when superposing protomer A onto protomer B. However, there is one notable difference, with protomer B displaying density for residues 206–221. These residues form part of a large loop insertion (LL1), which extends from Glu195 to Asn231 and includes Tyr229 of the MYW catalytic triad (Yamada et al., 2002; Regelsberger et al., 2001). However, density for these residues is not observed in protomer A (Figure S2A).

The structure of the heme environment of KatG^INH^ is shown in Figure 3C and is identical to this region in the X-ray crystal structure of WT KatG (Bertrand et al., 2004; Zhao et al., 2006) (Figure S2C), indicating that the inactivated pro-drug added to the sample is not perturbing the heme site. All particles of WT KatG^INH^ selected for the final structure classification contained a b-type heme in both protomers of the homodimer. The heme environment is structurally identical in both protomers and can be seen for protomer A in Figure 3C. The heme is pentacoordinate, with His270 coordinating the heme iron on the proximal side. Distal to the heme, well-defined density is observed, which is consistent with the previously determined covalent linkage between C^η2^ of Trp107 and C^ε1^ of Tyr229 and between C^ε2^ of Tyr229 and S^δ^ of Met255 to form the “MYW catalytic triad” (Figure 3D).

### INH binding to KatG

INH binding sites in several KatG homologs have been reported using X-ray crystallographic approaches or *in silico* modeling (Kamachi et al., 2015b; Wiseman et al., 2010; Marney et al., 2018). Our cryo-EM structure at 2.7 Å resolution is within the resolution range of the X-ray structures from which INH binding sites have been inferred (1.9–3.2 Å resolution) (Kamachi et al., 2015a, 2015b). Furthermore, cryo-EM is potentially preferable to X-ray crystallography for INH drug binding studies due to the risk of degradation of INH over long crystallization times and the potential crystal damage caused by soaking in INH.

**Figure 4:**
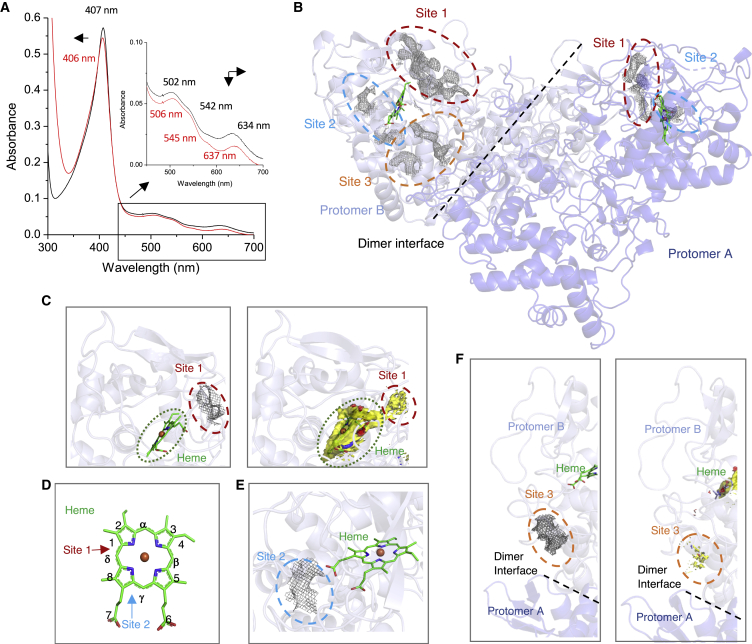
INH binding to KatG (A) UV-visible spectroscopy of WT KatG protein before and after the addition of INH. Black line shows WT resting state ferric KatG (6 μM) before the addition of INH and the red line after the addition of three INH equivalents. Inset shows the Q band region = and arrows indicate changes in wavelength and absorbance. All experiments were carried out at 20°C in 20 mM sodium phosphate, pH 7, 100 mM NaCl. (B) Sites of INH binding to WT KatG^INH^. Protomer A is shown in purple and protomer B in light purple. Extra density identified is shown as a gray mesh. The three areas of extra density are circled and colored red for site 1, blue for site 2, and orange for site 3. (C) Extra density for site 1 in protomer B near the heme corresponding to an identified hotspot (contour 14 cutoff, see the STAR methods). The hot spots are shown in yellow (hydrophobic), blue (hydrogen donor), and red (hydrogen acceptor), and the extra density for INH as a gray mesh. The heme is indicated by a green dashed circle and the binding site of INH as a red dashed circle. (D) Heme b nomenclature with sites 1 and 2 shown with arrows and labeled. (E) Extra density for site 2 in protomer B identified near the heme propionate groups. Site 2 is indicated with a blue dashed circle. (F) Enlarged site 3 hotspot (cutoff contour 14), with the hotspot shown in yellow (hydrophobic), blue (hydrogen donor), and red (hydrogen acceptor), and extra density from INH binding shown as a gray mesh with site 3 indicated with an orange dashed circle.

To confirm the binding of INH to KatG, we obtained electronic absorbance spectra of KatG in the presence and absence of INH (Figure 4A; Table S1). Purified WT KatG is brown in color and gives rise to transitions in the electronic absorbance spectrum. These electronic transitions arise from the bound b-type heme and generate wavelengths consistent with a resting state ferric catalase-peroxidase enzyme. Spectral transitions in the visible region comprise a Soret peak (arising primarily from π-π^∗^ transitions, common in proteins containing a coordinated heme) at 407 nm and charge transfer bands at 502 and 635 nm (Johnsson et al., 1997). After addition of INH, the Soret band and charge transfer bands display a decrease in absorbance concomitant with a wavelength shift indicative of INH binding (Figure 4A; Table S1).

The cryo-EM map of WT KatG before INH addition was compared with the WT KatG^INH^ structure to identify potential INH binding sites. Several areas of density that potentially correspond to INH were identified. In particular, we observed three potential binding site clusters rather than a homogeneous well-defined binding site (Figure 4B). Site 1 is present in both protomers and is located at the entrance to the heme pocket. Areas of extra density observed at site 1 are dispersed over a relatively large region that connects the bulk solvent to the heme site, giving the impression of INH binding along several locations within this solvent-accessible channel (Figures 4C and S3). To complement the structural data, a computational approach using the program Fragment Hotspot Maps was used (Radoux et al., 2016). This program looks for potential fragment binding sites in protein structural models. However, a limitation of the software is that a protein containing a cofactor, such as heme, results in a hotspot being identified in the heme binding cavity. Although there are regions corresponding to hydrogen donor and acceptor sites in both protomers, the hotspot is predominantly hydrophobic (yellow, Figures 4C and S3) and is unlikely to favor INH binding. Furthermore, within site 1 identified through cryo-EM, the Fragment Hotspot Maps program also identifies other hotspots with donor and acceptor regions in protomer A that would favor INH binding (Figures 4C and S3). This binding site is close to residues Ser315 and Asp137 toward the δ-edge of the heme (Figure 4D). This is of particular interest because Ser315 is one of the most prevalent mutation sites in INH-resistant strains (Munir et al., 2019; Isakova et al., 2018; Zhang et al., 2005). Site 2 is also present in both protomers, with density identified near the γ-edge of the heme (Figures 4D, 4E, and S3). This site is not detected using the Fragment Hotspot Maps program, indicating that this may be a weaker binding site for INH. However, some of the density identified within site 2 is in close proximity to the heme propionates. In APX L-ascorbate has been shown to bind close to heme propionate-6 (Metcalfe et al., 2007, 2008) (Figure 4D and S3). Finally, we identify density only in protomer B that could constitute a third binding site. This site is located toward the dimer-dimer interface and is within a large open pocket with part of this extra density in close proximity to a hotspot identified by the Fragment Hotspot Maps program (Figure 4F). While the density together with the bioinformatic analysis is indicative of INH binding sites we have not modeled discrete INH molecules. Rather we believe we are observing an ensemble of binding sites in keeping with a long-held view of how many small organic substrates interact with peroxidases (see Discussion).

### W107R and T275P KatG variants

**Figure 5:**
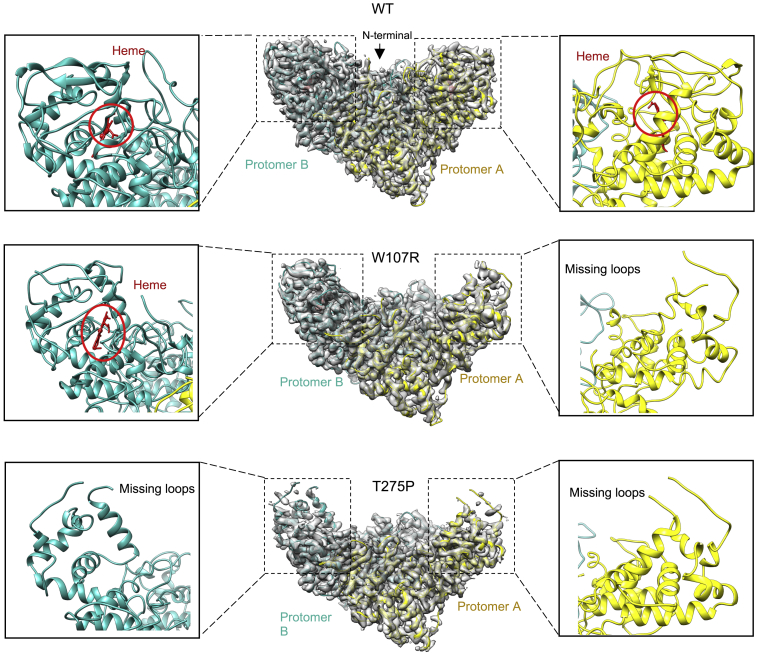
Cryo-EM structures of WT^INH^, W107R, and T275P KatG proteins from *M*. *tuberculosis* The cryo-EM maps of WT^INH^, W107R, and T275P as purified KatG proteins with the density shown in gray, the protomer A main chain in yellow, and the protomer B main chain in cyan. Insets show enlarged regions to indicate the presence or absence of heme and loop regions. Heme is shown in red and indicated by a red circle.

Having developed a protocol for structural studies of WT KatG, we selected two clinically relevant mutants to establish whether our methodology could also be applied to KatG variants; W107R and T275P. Trp107 is a catalytic residue, whereas Thr275 is on a loop close to the heme binding site. For the W107R variant, the best class produced a cryo-EM map to 3.3 Å resolution (Figure 5). This model surprisingly displays only one heme bound per homodimer of protein (Figure 5). The heme is absent from protomer A and displays significant structural disorder in the vicinity of the heme binding site. Several areas surrounding the heme pocket were difficult to model in protomer A, either displaying minimal or fragmented density (Figure 5, inset). In contrast, the C-terminal domain of both protomers remains similar to the WT KatG. The resolution of the map is sufficient to enable an Arg residue to be modeled in place of Trp107 in protomer B, resulting in disruption of the covalently linked catalytic triad (Figure S4). The remaining residues within the heme site are superposable with the WT protein (Figure S4). Furthermore, the loop containing Tyr229, which is part of the MYW catalytic triad is disordered in both protomers, presumably as a consequence of the mutation.

A cryo-EM map to 3.4 Å resolution was obtained for the T275P variant (Figure 5). The structure again displays significant areas of disorder compared with the WT KatG. Several loops surrounding the heme pocket contain little or no density in either protomer A or B. These disordered regions are identical to those in protomer A of the W107R variant (see above). The loop containing the Thr275 residue (residues 274–329) displays no density and therefore could not be modeled. Local resolution maps and Fourier shell correlation (FSC) curves of W107R and T275P can be seen in Figure S5. Likewise, fragmented density within the heme pocket was not sufficient to satisfactorily model a heme in either protomers. In addition, other heme pocket residues, such as Trp107 and His270, are significantly perturbed compared with the WT KatG structure. Furthermore, no density was observed for the loop containing Tyr229, a residue that is part of the MYW catalytic triad, thus suggesting that the crosslink is not present in this KatG variant.

### Heme content and peroxidase activity of the W107R variant

**Figure 6:**
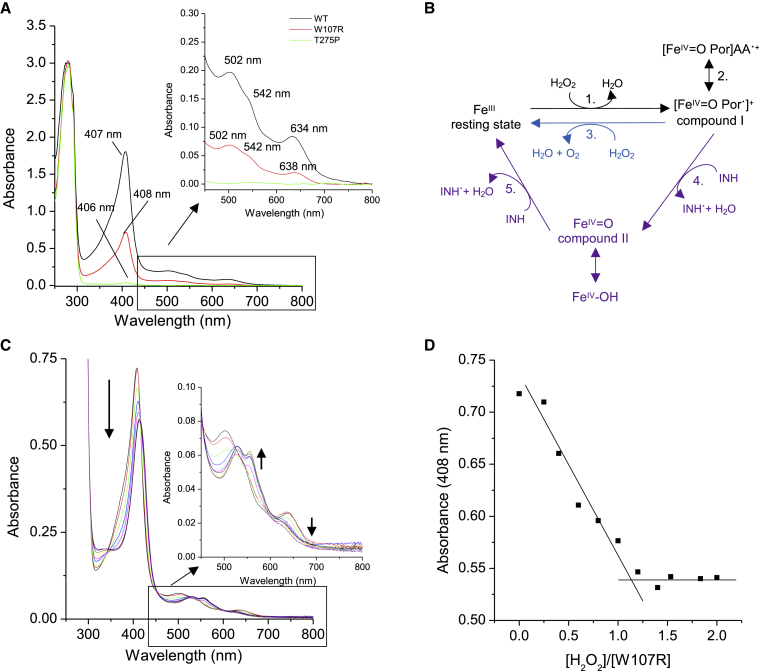
UV-visible spectra of KatG proteins and peroxidase activity of the W107R KatG protein from *M*. *tuberculosis* (A) UV-visible absorbance spectrum of the purified T275P (green) and W107R (red) variants compared with WT (black) KatG, with the Soret band labeled. Inset displays theQ bands region with the absorbance maxima labeled. (B) Reaction scheme for KatG and other heme catalases and peroxidases with INH shown as the oxidizing substrate. Each reaction is numbered, and reactions performed by only catalases are shown in blue and only by peroxidases in purple. (C) Changes in the UV-visible spectrum of the W107R upon addition of H_2_O_2_. Inset shows the Q band region region with arrows indicating the direction of absorbance maxima after adding H_2_O_2_. (D) The absorbance change at 408 nm for the W107R variant plotted against the ratio of H_2_O_2_ to protein concentration. The intersection of the solid lines indicate an approximate 1:1 (H_2_O_2_:protein) stoichiometry. All experiments carried out at 20°C in 20 mM sodium phosphate, pH 7, 100 mM NaCl.

Having noticed from our cryo-EM maps the loss of heme in both variants, we sought to assess spectroscopically the heme content of the variant proteins. Purified WT KatG has a *Reinheitszahl* ratio (R_z_ = A_407_/A_280_) of 0.53–0.69 (Wengenack et al., 2000; Yu et al., 2003; Ghiladi et al., 2005) consistent with the protein being fully loaded with heme (Figure 6A). Our cryo-EM structure of KatG^INH^ is in agreement with this observation, as a heme is clearly defined within both protomers (it is important to note that 2-fold symmetry averaging is not imposed on this map) (Figure 2). Interestingly, despite the same growth conditions as the WT KatG, using the heme supplements, aminolevulinic acid and hemin chloride, the two KatG variants, W107R and T275P, gave R_z_ values of 0.2–0.3 and 0.003–0.007, respectively, indicating a lower heme occupancy (Figure 6A; Table S1). Using the pyridine hemochromogen assay, the percentage of b-type heme within the three purified proteins was on the order of WT (98%) > W107R (31%) > T275P (3.5%). The Soret peak and charge transfer bands of the W107R variant are essentially identical to those of the WT enzyme, suggesting that despite the lower heme content the electronic structure of the heme is not perturbed. This is in agreement with our W107R cryo-EM structure, which shows only one heme bound, but in an arrangement identical to that in the WT KatG.

The W107R variant is predicted to disrupt the MYW catalytic triad in the distal heme site. It has been reported that disruption of this catalytic triad leads to an absence of catalase activity (Cade et al., 2010). The generalized reaction scheme for catalase-peroxidases can be seen in Figure 6B. The first step in the catalase and peroxidase reactions is common to both, where, upon addition of H_2_O_2_ to the Fe^III^ (ferric) enzyme, the heme becomes oxidized to form compound I ([Fe^IV^ = O Por^⋅^]^+^) (1. in Figure 6B) or a protein radical is formed by intramolecular electron transfer ([Fe^IV^ = O Por]AA^⋅+^) (2. in Figure 6B). The catalase and peroxidase reactions then differ in their reduction of compound I back to the resting Fe^III^ state. The catalase reaction involves a single two-electron transfer reaction from H_2_O_2_ (3. in Figure 6B), whereas the peroxidatic reduction of compound I involves two consecutive one-electron transfer steps. First, compound I is reduced to compound II (Fe^IV^ = O or Fe^IV^-OH) (4. in Figure 6B) followed by reduction to Fe^III^ via two single-electron reduction reactions (5. in Figure 6B). Titrating H_2_O_2_ into the WT KatG and the W107R variant leads to the spectral transitions reported in Figures S6 and 6C. For WT KatG, no heme species intermediates, i.e., compound I or compound II, were spectrally observed on addition of 2-fold excess of H_2_O_2_. As has previously been observed using rapid mixing conditions, these heme intermediates form and decay on a millisecond timescale and, thus, the absence of changes under our conditions is indicative of catalase activity being present in this enzyme. In contrast, for the W107R variant spectral transitions are observed (Figure 6C), resulting in an endpoint spectrum that resembles that of a peroxidase compound II species. The titration reveals a stoichiometry of 1:1 (W107R:H_2_O_2_) with no further spectral changes, implying that the W107R variant retains peroxidase activity but not catalase activity (Figure 6D). According to the peroxidase mechanism for KatG the first catalytic intermediate is a compound I species that would form after a stoichiometric addition of H_2_O_2_. However, as documented with other peroxidases, compound I rapidly reduces to compound II owing to internal electron transfer, as implied from our titration (Figure 6D; Table S1).

In contrast, although the T275P variant displays a Soret band at a wavelength similar to the WT and W107R variant, the charge transfer bands, while weak, do not coincide with those observed for the WT and W107R variant (Figure 6A; Table S1). In our cryo-EM structure for this variant we did not see any ordered heme bound, again consistent with the spectroscopic data. Owing to the low heme content (3.5%) similar H_2_O_2_ titration experiments with the T275P were not conducted.

### Heme uptake studies

**Figure 7:**
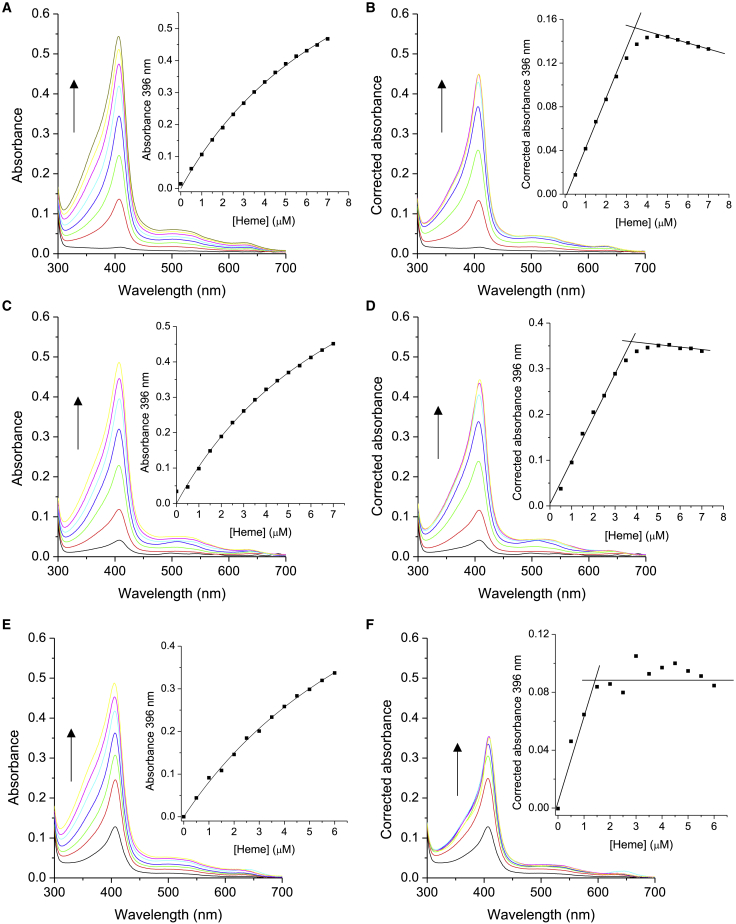
Addition of hemin chloride to T275P, WT, and W107R KatG protein (A) Changes in the UV-visible spectrum of the T275P variant (3.3 μM) upon the addition of hemin chloride. The arrow indicates the absorbance increase upon addition of hemin chloride. Inset displays the absorbance at 396 nm plotted against heme concentration with a hyperbolic fit. (B) Corrected UV-visible absorbance spectrum of T275P KatG variant protein (see the STAR methods) following the addition of hemin chloride. Inset displays the corrected absorbance at 396 nm plotted against heme concentration with lines to indicate stoichiometry. The downward slope at higher free heme concentrations is due to the non-linearity of the absorbance of heme as a function of concentration (see the STAR methods and Figure S6). (C) WT KatG protein identical to (A). (D) Corrected UV-visible absorbance spectrum of WT KatG identical to (B). (E) W107R KatG variant protein identical to (A and C). (F) Corrected UV-visible absorbance spectrum of W107R KatG variant protein identical to (B and D). All at 20°C in 20 mM NaPi, pH 7, 100 mM NaCl.

Both solution data and cryo-EM structures, described here, indicate that heme occupancy is low in both variants. We next assessed whether the variants still retained the ability to bind heme if it were added exogenously. Upon titration of hemin chloride (in concentrations similar to that encountered *in vivo*) to the variants, absorbance increases were observed in the Soret and visible regions of the electronic absorbance spectrum (Figures 7A, 7C, and 7E). Assessing heme binding is complicated because of the difficulty in discriminating between the Soret absorbance peak for the holo-protein and the titrant (hemin), which also gives rise to spectral transitions in this region (see Figure S7). To address this complication, we devised a method that enables discrimination between the contribution of protein-bound heme and free heme to the absorbance spectrum at any given heme concentration (see the STAR methods). Using this approach, titration curves that report only on protein-bound heme can be plotted as illustrated in Figures 7B, 7D, and 7F. For comparison, we also included titration of heme into the WT KatG, using a sample in which the heme had been removed (see the STAR methods) (Figure 7B). The plots in Figures 7B, 7D, and 7F reveal a linear increase in absorbance at 396 nm, until a break point is reached with no further changes in absorbance. Under the titration conditions used (μM protein concentration), these plots are representative of the stoichiometry of heme binding and preclude the determination of heme binding affinity, which can be estimated to be in the sub-micromolar range. For the WT protein and the T275P variant, the plots indicate a heme protein stoichiometry of 1:1 (Figures 7B and 7D). However, for the W107R variant the stoichiometry determined is ∼0.5:1, which would be expected based on the pyridine hemochromagen assay that predicts ∼30% heme occupancy (Figure 7F).

These results indicate that heme can be fully loaded into both variants. Therefore, it would be expected that the pyridine hemochromagen assay would confirm the observations from the heme titrations. Samples from the titrations were passed through a small desalting column to remove excess heme before the pyridine hemochromagen assay. Surprisingly, the values determined do not show 100% occupancy, but rather 66% and 56%, for the W107R and T275P variants, respectively. This suggests that, although exogenous heme is readily taken up by the variants, in the absence of free heme in solution it dissociates within the column transit time and in the time before the assay. Such heme dissociation does not occur with the WT protein.

### Cryo-EM structures of the W107R and T275P variants after heme uptake

**Figure 8:**
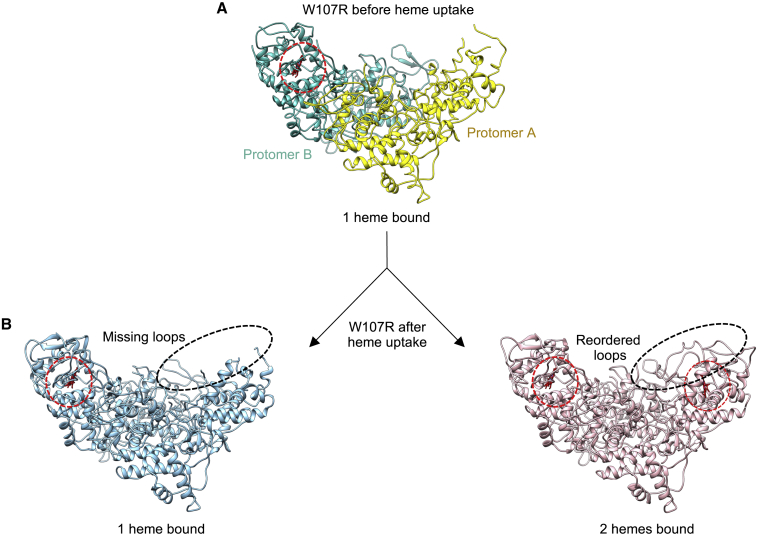
Cryo-EM structures of the W107R variant KatG protein before and after heme loading (A) The structure of W107R as purified, protomer A in yellow and protomer B in cyan. (B) The two structures of W107R following heme loading. Left, the structure of W107R with one heme molecule bound from the 3.2 Å resolution map. Right, the structure of W107R with two heme molecules bound from the 3.1 Å resolution map. Heme molecules are shown in red and highlighted with a dashed red circle. Missing and reordered loops are also shown with a dashed black line.

Having established that the two variants could be loaded with exogenous heme, we obtained cryo-EM structures of the proteins following heme uptake, to compare them with those already obtained before heme loading. For the W107R variant, two structures were solved to 3.2 and 3.1 Å resolution, respectively. The structure solved to 3.2 Å resolution was essentially identical to the W107R structure before heme loading (Figure 8B). In this structure the heme was again only identified in protomer B and was absent from protomer A (Figure 8B, left). However, in the 3.1 Å resolution structure, heme could be modeled into well-defined density present in both protomers (Figure 8, right). The presence of heme in protomer A coincides with the reordering of the loop structures in its immediate vicinity, thus indicating the importance of heme binding for structural order in this variant.

For the T275P variant, after the heme titration the majority of the particles were assigned into a class that produced a cryo-EM map to 3.6 Å resolution. Notably, this structure is identical to the T275P variant structure before heme loading (0.7 Å RMSD for 551 Cα atoms in protomer A and 0.8 Å RMSD for 542 Cα atoms in protomer B) (Figure S8), with no well-defined density that enables heme to be modeled in either protomer. The lack of heme density is thus in line with our observation that heme is more easily lost in this variant. Notably, the only difference between the two structures is in helix 238–251, which moves toward the heme environment after heme is loaded into this variant (Figure S8). FSC curves and local resolutions for W107R and T275P following heme loading are shown in Figure S9.

## Discussion

INH binding to KatG and its subsequent activation has been the focus of many studies using a variety of techniques, such as stopped-flow kinetics, X-ray crystallography, and electron paramagnetic resonance spectroscopy. In this study we have applied cryo-EM to demonstrate the feasibility of use of using this technique to aid the identification of small-molecule binding regions. Cryo-EM has been previously used to study drug binding to macromolecular assemblies; for example, the structure of the cytoplasmic ribosome from *Plasmodium falciparum* in complex with an anti-protozoan drug (Wong et al., 2014). The same methodology may also be more widely applicable to include relatively low-molecular-weight proteins and drug molecules, as we have now demonstrated with KatG (∼161 kDa) and INH (∼137 Da). Furthermore, our study demonstrates that cryo-EM is able to identify an ensemble of transient drug binding sites.

KatG is not unique in its ability to turn over INH and related molecules; the class I peroxidases C*c*P and APX, and the class III peroxidase HRPC, can also perform this role (Pierattelli et al., 2004; Shoeb et al., 1985). Crystallography has revealed that in both APX and C*c*P, INH can bind in the proximity of the heme δ-edge (Metcalfe et al., 2008). Binding of INH at the δ-edge of the heme in HRPC has also been demonstrated in solution using NMR spectroscopy (Pierattelli et al., 2004). It has also been predicted that INH binds to *M*. *tuberculosis* KatG in a similar manner as to HRPC, APX, and C*c*P (Bertrand et al., 2004; Metcalfe et al., 2008). In agreement with these observations we identify site 1 in our KatG^INH^ structure (Figure 4C). Although the extra density present in site 1 is not clearly defined, our computational approach corroborates this to be a binding hotspot (Figure 4C). This site is located toward the entrance of the distal heme pocket near residues Ser315 and Asp137, with both of these residues previously reported to regulate INH activation (Zhao et al., 2013; Pierattelli et al., 2004).

Two further potential binding sites (2 and 3) were identified from the cryo-EM structures (Figure 4). All three sites observed are almost identical in location to the sites reported for the WT KatG structure from *Synechococcus elongatus* (PDB: 3WXO; Kamachi et al., 2015a). Our site 2 is located near the γ-heme edge and is consistent with the second INH binding site reported for APX (Metcalfe et al., 2007, 2008). The third INH binding site (site 3) identified from our cryo-EM data also correlates with site 3 from *S*. *elongatus* KatG. This site is close to Gly299 and Trp300, residues for which mutations have been identified, and reported to be associated with INH resistance in clinical isolates (Cade et al., 2010; Torres et al., 2015a; Vilcheze and Jacobs, 2014). Therefore, the extra density we observe in our cryo-EM KatG^INH^ structure aligns well with previous X-ray crystallography studies of INH binding.

Previous attempts to obtain crystal structures of KatG bound to INH have proven unsuccessful (Zhao et al., 2006). It may therefore not be surprising in our cryo-EM KatG^INH^ structure that we do not observe discrete well-defined density to model a single INH molecule. Instead we observe several regions of extra density. An explanation for this could be that the binding of INH is a transient and dynamic interaction, resulting in promiscuity within a broadly distributed binding site. For many peroxidases, their natural substrates are not known, yet they display a wide-ranging specificity with small organic molecules that can be readily oxidized (Dunford, 2010). INH undergoes a very rapid oxidation mechanism with KatG (Chouchane et al., 2000) and thus a view that may be advanced is that INH need only be in proximity of the heme for rapid electron transfer to occur. In view of this explanation, and our experimental data, we propose that INH enters the heme pocket via site 1, where the largest area of extra density is observed and has the highest hotspot prediction. Once in the site, INH samples multiple orientations of which some will be productive. Therefore, our data may be considered as an average of multiple possible binding orientations.

Difficulty in the ability to produce consistently crystals of KatG variants led us to use cryo-EM for investigating the structural impact of resistance mutations. While it has been established that the T275P variant of KatG is associated with a high-level INH resistance (MIC >10) (Pym et al., 2002), the effect of the mutation on catalase and/or peroxidase activity is less clear. No detectable catalase activity has been reported, whereas for peroxidase activity contradictory reports of activity are noted (Pym et al., 2002; Cade et al., 2010). This protein variant was found to express in very low quantities in both *E*. *coli* and in an *M*. *tuberculosis* mouse model (Saint-Joanis et al., 1999; Pym et al., 2002). We also obtained lower expression levels in *E*. *coli* compared with the WT protein and perhaps more importantly observed a significant decrease in the percentage of heme uptake during expression and purification (∼99% in WT compared with 3.5% in the T275P variant). The cryo-EM structure of the T275P variant reveals a considerably disordered structure compared with the WT protein. Several loop regions surrounding the heme pocket that are well defined in the WT protein cannot be modeled owing to the absence of density. The MYW catalytic triad is no longer formed and we observe no density for the Tyr229 residue (a residue part of the MYW triad). The MYW crosslink has been shown previously to be important in the catalase activity of the protein and thus our observation that this crosslink is no longer present would indicate that this variant no longer has catalase activity. As expected from our heme binding assays, the structure of the T275P variant before heme loading displayed little density for a well-defined bound heme. Despite absorbance spectroscopy clearly indicating heme uptake in this variant, the cryo-EM structure obtained after heme loading again showed a lack of defined density for a coordinated heme. Therefore, although the T275P variant is able to readily take up exogenous heme, in a similar manner to WT KatG (Figure 7) it is likely that the heme dissociation rate is high, meaning that heme is not retained. Without correctly incorporated heme, this variant, together with the absence of the MYW crosslink, is unlikely to be able to function as a catalase-peroxidase (Pym et al., 2002; Cade et al., 2010). The ability of cryo-EM to capture these disordered states driven by the presence or absence of heme provides an explanation as to why this variant leads to INH resistance.

For the W107R variant, the as-purified cryo-EM structure also revealed heme absence and disorder in regions associated with heme binding (Figure 5). Thus, as with the T275P variant, the inability to fully complement with heme is a common feature in these resistance variants. However, unlike the T275P variant, the W107R has a higher capability to retain bound heme, as demonstrated from its presence and reordering of the disordered regions in the cryo-EM structure obtained following addition of exogenous heme (Figure 8).

In summary, our study has indicated the presence of several potential binding sites for the small INH pro-drug, which is a fragment-sized molecule where promiscuous binding is expected. Although it has been a challenge to identify the functional state of INH, this study does highlight the power of using cryo-EM to visualize dynamic small-molecule binding and structural disorder induced by mutations that lead to resistance. This study sets the basis for understanding any KatG resistance mutation and demonstrates that cryo-EM could be helpful in understanding the impacts of mutations in a broad range of biological systems.

## STAR★Methods

### Key resources table

**Table:** 

REAGENT or RESOURCE	SOURCE	IDENTIFIER
**Bacterial and Virus Strains**		

B21(DE3) *E*. *coli*	Lab stock	N/A

**Chemicals, Peptides, and Recombinant Proteins**		

KatG recombinant protein	Lab	N/A
Hemin chloride	Sigma-Aldrich	H9039
5-Aminolevulinic acid hydrochloride	Sigma-Aldrich	A3785
Hydrogen Peroxide Solution	Sigma-Aldrich	H1009

**Deposited Data**		

WT KatG structure	This paper	PDB: 6ZJI
WT KatG Cryo-EM map	This paper	EMDB: 11234
WT KatG +INH structure	This paper	PDB: 7AG8
WT KatG +INH Cryo-EM map	This paper	EMDB: 11776
T275P structure	This paper	PDB: 7A8Z
T275P Cryo-EM map	This paper	EMDB: 11680
T275P +Heme structure	This paper	PDB: 7AA3
T275P +Heme Cryo-EM map	This paper	EMDB: 11689
W107R structure	This paper	PDB: 7A2I
W107R Cryo-EM map	This paper	EMDB: 11625
W107R + Heme (1 heme) structure	This paper	PDB: 7A7C
W107R + Heme (1 heme) Cryo-EM map	This paper	EMDB: 11677
W107R + Heme (2 heme) structure	This paper	PDB: 7A7A
W107R + Heme (2 heme) Cryo-EM map	This paper	EMDB: 11676
The crystal structure of KatG from *Mycobacterium tuberculosis*.	Zhao et al., 2006	PDB: 2CCA
The crystal structure of Isoniazid bound KatG from *Synechococcus elongatus*.	Kamachi et al., 2015a, 2015b	PDB: 3WXO

**Oligonucleotides**		

T275P KatG Forward primer 5’-ACCTTTGGTAAACCACATGGTGCAGGT-3’	This paper	N/A
T275P KatG Reverse primer 5’-ACCTGCACCATGTGGTTTACCAAAGGT-3’	This paper	N/A
W107R KatG Forward primer 5’-TGCTGCATGCCGTGCCATACGAATAAACAGCGG-3’	This paper	N/A
W107R KatG Reverse primer 5’-CCGCTGTTTATTCGTATGGCACGGCATGCAGCA-3’	This paper	N/A

**Recombinant DNA**		

W107R – pET28a plasmid	This paper	N/A
T275P – pET28a plasmid	This paper	N/A
WT – pET28a plasmid	This paper	N/A

**Software and Algorithms**		

Warp	Tegunov and Cramer, 2019	http://www.warpem.com/warp/
CryoSPARC	Punjani et al., 2017a, Punjani et al., 2017b	https://cryosparc.com
Coot	Emsley et al., 2010	https://www2.mrc-lmb.cam.ac.uk/personal/pemsley/coot/
PHENIX Real-space refinement	Afonine et al., 2018b	https://www.phenix-online.org/documentation/reference/real_space_refine.html
Fragment Hotspot Maps	Radoux et al., 2016	http://fragment-hotspot-maps.ccdc.cam.ac.uk/
Namdinator	Kidmose et al., 2019	https://namdinator.au.dk
UCSF chimera	Pettersen et al., 2004	https://www.cgl.ucsf.edu/chimera/download.html

### Resource availability

#### Lead contact

Further information and requests for resources and reagents should be directed to and will be fulfilled by the Lead Contact, Amanda K Chaplin (ac821@cam.ac.uk).

#### Materials availability

Wild-type or mutant expression plasmids are available upon request. This study did not generate new unique reagents.

#### Data and code availability

Data supporting the findings of this manuscript are available from the corresponding authors upon reasonable request. All data generated or analysed during this study are included in this published article (and its supplementary information files). Cryo-EM density maps have been deposited in the Electron Microscopy Data Bank with accession codes EMD-11234, 11776, 11625, 11677, 11676, 11680 and 11689. Atomic coordinates have been deposited in the RCSB Protein Data Bank with accession codes PDB 6ZJI, 7AG8, 7A2I, 7A7C, 7A7A, 7A8Z and 7AA3 (Table 1).

### Experimental model and subject details

BL21(DE3) *E*. *coli* cells grown at 37°C then reduced to 18°C following induction of protein expression.

### Method details

Cloning, expression and purification of WT, W107R and T275P KatG

The synthetic construct of wildtype (WT) KatG in plasmid (pMAT) was ordered from Thermo fisher and transferred to the pHAT4 plasmid (having a His-tag at the N-terminal). The KatG mutants W107R and T275P were prepared using site-directed mutagenesis with the primers shown below:

T275P

5’-ACCTTTGGTAAACCACATGGTGCAGGT-3’

5’-ACCTGCACCATGTGGTTTACCAAAGGT-3’

W107R

5’-TGCTGCATGCCGTGCCATACGAATAAACAGCGG-3’

5’-CCGCTGTTTATTCGTATGGCACGGCATGCAGCA-3’

All constructs of KatG were expressed in *E*. *coli* BL21(DE3) cells. 250 ml of primary culture was grown overnight at 37°C, which was then used to inoculate 6 x 1L Erlenmeyer flasks of 2XYT media and incubated at 37°C until an OD600 of 0.5 was reached. Just before induction, aminolevulinic acid at a final concentration of 300 μM was added to the media and the cultures were subsequently induced with 500 μM of IPTG and incubated overnight at 18°C. The cells were then harvested by centrifugation at 3,501 g for 30 min at 4°C. Both the WT KatG protein and the variants were purified based on the protocol of (Marcinkeviciene et al., 1995) Marcinkeviciene et al*.*, 1995 (Marcinkeviciene et al., 1995) and Zhao et al*.*, 2006 (Zhao et al., 2006), with slight modifications as described in detail below.

The cells were resuspended in lysis buffer containing: 20 mM potassium phosphate, pH 7.2, 20 mM Imidazole, 500 mM NaCl, 5 mM MgCl_2_, Protease Inhibitors and DNase I. Cells were then incubated with stirring at 4°C for an hour after the addition of 100 μM (final concentration) of hemin chloride. They were lysed with sonication and incubated again at 4°C with stirring for 30 min. The cell debris was removed by centrifugation at 38,724 g for 20 min at 4°C and the supernatant filtered. The clarified supernatant was passed through the IMAC (nickel-sepharose) column equilibrated with lysis buffer. Proteins were eluted using Elution Buffer (500 mM Imidazole, 20 mM Potassium phosphate pH 7.2, 500 mM NaCl). TEV protease was added to remove the His-tag. The protein was dialysed overnight in 20 mM potassium phosphate, pH 7.2 and subjected to the reverse IMAC to remove the His-tag. The protein was loaded onto the Hi-trap Q anion exchange column equilibrated with 20 mM potassium phosphate buffer pH 7.2 and eluted using a 0-1 M NaCl gradient. The eluted fractions were pooled and concentrated to 5 ml and applied to the Superdex S200 gel filtration column equilibrated with 20 mM potassium phosphate, pH 7.2. The fractions eluted were pooled and applied to the MonoQ anion exchange column and the protein eluted using 0-1 M NaCl gradient. The eluted fractions were pooled, and ammonium sulphate added to a final concentration of 1M. Protein was loaded onto a phenyl-sepharose column equilibrated with 20 mM potassium phosphate buffer containing 1 M ammonium sulphate. Proteins were eluted using a reverse gradient of 1-0 M ammonium sulphate in 20 mM potassium phosphate, pH 7.2.

#### UV-visible spectroscopy of KatG

All UV-visible spectra were recorded at 20°C in 20 mM NaPi, pH 7, 100 mM NaCl. Concentrations of WT KatG and the two variants were determined using a Cary 60 UV-visible spectrophotometer (Agilent), with a 1 cm path-length Quartz cuvette (Hellma). The absorbance at 280 nm was measured and concentrations calculated using the Beer-Lambert law with an extinction coefficient (ε) of 163,290 M^-1^ cm^-1^ for WT and T275P KatG, and 157,790 M^-1^ cm^-1^ for W107R KatG variant determined using ProtParam ExPASY. Hydrogen peroxide (H_2_O_2_) solutions (Sigma) were prepared by diluting stocks using deionised water and concentrations determined using ε of 43.6 M^-1^ cm^-1^ at 240 nm (Beers and Sizer, 1952). Concentrations of H_2_O_2_ were used to check the peroxidase activity of WT KatG and lack of activity with W107R. Stock solutions of hemin chloride were prepared by dissolving the solid in 1 ml Milli-Q water with the addition of 2 μl of 10 M NaOH. Isoniazid (INH) (Sigma) was prepared by dissolving solid power in 20 mM potassium phosphate, pH 7.

#### Hydrogen peroxide and INH binding to KatG

All titration experiments were carried out using a Cary 60 UV-visible spectrophotometer (Agilent) at 20 ^o^C in 20 mM NaPi, pH 7, 100 mM NaCl buffer. H_2_O_2_ was prepared as above and titrated into the resting state ferric WT and W107R proteins until no further changed was detected. The absorbance change at 408 nm for W107R was plotted against a ratio of [H_2_O_2_]/[W107R] to determine the stoichiometry of binding. INH was prepared as described above and titrated into WT KatG protein to observe spectra changes.

#### Heme binding to KatG

Hemin chloride was prepared as described above and titrated into WT, W107R and T275P as well as a control titration into 20 mM potassium phosphate, pH 7 buffer. Deconvolution of the spectral contributions of free heme and reconstituted KatG was effected using a simple protocol that is based on measuring the ratio of absorbances at two selected wavelengths after each heme addition. This ratio is then compared with the ratios determined at the identical wavelengths for the individual components, free heme and KatG. The wavelengths selected were 350 nm and 396 nm, the wavelength of the distinctive shoulder and the peak of the free heme, respectively, see spectrum Figures S7 and 7.

If we define the ratio (A_396_/A_350_) as r_1_ for KatG, as r_2_ for free heme and as r_3_ for any selected spectrum collected during the titration then it may be shown that

(r_1_-r_3_)/(r_3_-r_2_) = (x/y)_350nm_

where x is the absorbance of free heme at 350 nm and y the absorbance of reconstituted KatG at 350 nm.

We know the absorbance value at 350 nm and this is equal to x+y and thus we can calculate the individual contributions x and y. In these experiments we are interested to know y, the spectral contribution of KatG as we add free heme. Once y is known the contribution at all wavelengths and hence the full spectrum at each point in the titration may be determined.

This method assumes that the spectral contribution of heme bound adventitiously to the protein is negligible. A confounding issue is that free heme in buffer does not obey the Beer Lambert Law. As seen in Figure S7 the apparent extinction coefficient of free heme decreases at higher concentrations (due to heme-heme association), We have selected the spectrum collected at 2 μM heme to calculate the ratio r_2_. This choice was made on the basis that up to that concentration the extinction coefficient is approximately constant. Also, in the titrations the concentration of free heme only exceeds 2 μM in the latter portion of the titration. In this region of the titration the protocol delivers a value of y that is too small because the contribution of free heme is overestimated and hence we observe in the plots of A_396_ versus [heme] a downward slope of the plot at high [heme].

Although the protocol described has deficiencies it nevertheless provides clear evidence of heme insertion into the proteins and yields satisfactory estimates of stoichiometries and upper limits for binding constants.

#### Pyridine hemochromagen assay

Protoheme content was measured by the pyridine hemochrome assay according to Barr and Guo using Δε_557_ = 20.7 mM^–1^cm^–1^ (reduced minus oxidized) for iron protoporphyrin IX (Barr and Guo, 2015).

#### Cryo-EM grid preparation

Purified WT, W107R or T275P with and without heme loading KatG protein samples were mixed with 8 mM CHAPSO (3-[(3-Cholamidopropyl)dimethylammonio]-1-propanesulfonate) (final concentration) prior to 3 μl of the protein between 3-4 mg/ml being applied to glow discharged for 60 sec at current of 25 mA in PELCO Easiglow (Ted Pella, Inc) holey carbon grids (Quantifoil Cu R1.2/1.3, 300 mesh). WT KatG with INH was prepared by adding INH (30 mM) directly before loading the protein to the grids to prevent degradation of INH. The optimal concentration of 30 mM INH was determined as lower concentrations did not display extra density for INH and higher concentrations gave a high level of background, leading to low quality data. The grids were then blotted with filter paper once to remove any excess sample, and plunge-frozen in liquid ethane using a FEI Vitrobot Mark IV (Thermo Fisher Scientific Ltd) at 4°C and 95 % humidity.

#### Cryo-EM data acquisition

All cryo-EM data presented here were collected in the Department of Biochemistry, University of Cambridge and all data collection parameters are given in Table 1.

#### Image processing

Data were processed using Warp (Tegunov and Cramer, 2019) and CryoSPARC (Punjani et al., 2017a, 2017b). In short, CTF correction, motion correction, and particle picking were performed using Warp. These particles were subjected to two-dimensional (2D) classification in CryoSPARC followed by *ab initio* reconstruction to generate initial 3D models. Particles corresponding to different classes were selected and optimised through iterative rounds of heterogeneous refinement as implemented in CryoSPARC. The best models were then further refined using homogenous refinement and finally non-uniform refinement in CryoSPARC. The final reconstructions obtained had overall resolutions (Table 1), which were calculated by Fourier shell correlation at 0.143 cut-off.

#### Structure refinement and model building

The final cryo-EM maps were used for model building. The model from the X-ray crystal structure of WT KatG (PDB 2CCA) was used as an initial template and rigid-body fitted into the cryo-EM density in UCSF chimera (Pettersen et al., 2004) and manually adjusted and rebuilt in Coot (Emsley et al., 2010). Namdinator (Kidmose et al., 2019) was used to adjust the structure and several rounds of real space refinement were then performed in PHENIX (Afonine et al., 2018b) before the final model was validated using Molprobity (Afonine et al., 2018a). Following refinement of the WT KatG structure the variants were also modelled using Namdinator (Kidmose et al., 2019) followed by refinement using Coot (Emsley et al., 2010) and PHENIX real space refinement (Afonine et al., 2018b). All structures we refined and validated before being deposited into the PDB and EMDB with accession codes given in Table 1.

#### Hotspot analysis

Fragment Hotspot Maps (Radoux et al., 2016) is a software for identifying interactions that determine fragment binding on protein hotspots. The webserver, available at http://fragment-hotspot-maps.ccdc.cam.ac.uk/, was used to run the hotspot calculations on KatG. A cut-off of 14 was used and the hotspots compared to the extra density identified in the INH bound KatG structure.

### Quantification and statistical analysis

All cryo-EM data were processed using Warp (Tegunov and Cramer, 2019) and CryoSPARC (Punjani et al., 2017a, 2017b) and refined using coot (Emsley et al., 2010) and PHENIX real space refinement (Afonine et al., 2018b). All structure statistics can be found in Table 1.

## Supplemental information

Document S1. Figures S1–S9 and Table S1

Document S2. Article plus Supplemental information
