# Supplementary material for: Using cryo-EM to understand antimycobacterial resistance in the catalase-peroxidase (KatG) from Mycobacterium tuberculosis
Source: Structure. 2021 Aug 5;29(8):899–912.e4. doi: 10.1016/j.str.2020.12.008 (PMC8355310; doi:10.1016/j.str.2020.12.008)
Supplement: Document S1. Figures S1–S9 and Table S1 [file mmc1.pdf]

Structure, Volume *29*

Supplemental Information

Using cryo-EM to understand antimycobacterial resistance in the catalase-peroxidase (KatG) from *Mycobacterium tuberculosis*

Asma Munir, Michael T. Wilson, Steven W. Hardwick, Dimitri Y. Chirgadze, Jonathan A.R. Worrall, Tom L. Blundell, and Amanda K. Chaplin

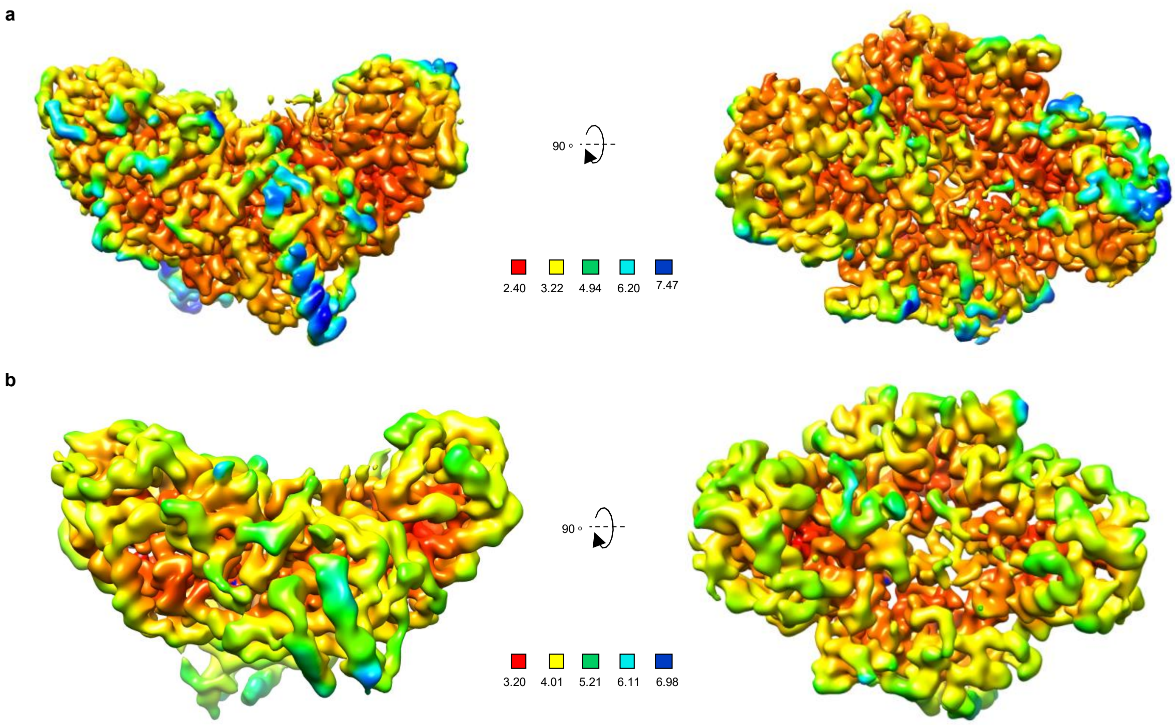

Figure S1: Local resolution (Å) **- maps of (a) WT KatG and (b) WT KatG^{INH}.** The inset beneath each structure indicates the colour of the map with the corresponding resolution. Related to Figure 2.

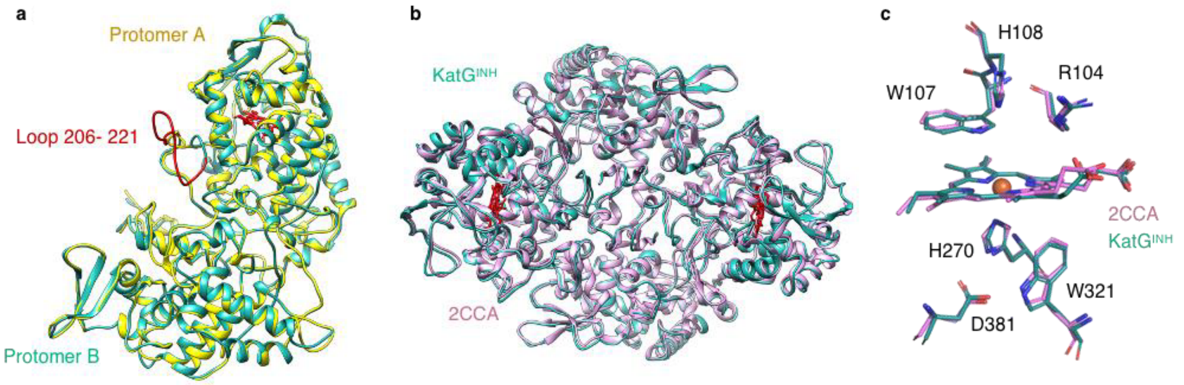

Figure S2: Cryo-EM model of WT KatG from *M. tuberculosis*. a) Comparison between protomer A and protomer B from our WT KatG^{INH} model. Protomer A is shown in yellow and protomer B in cyan. The heme in both protomers and the loop region present in only protomer B (residues 206-221) are

shown in red. **b and c)** Comparison between our WT KatG model and the model from PDB entry 2CCA (Zhao et al., 2006). 2CCA is shown in pink and our model in cyan**. b)** compares the overall structure and **c)** shows a comparison of the heme active sites with residues labelled. Related to Figure and 3.

Table S1: UV-visible absorbance maxima of WT, W107R and T275P. (*sh*) stands for shoulder. Related to Figure 4.

WT	WT + INH	W107R	W107R + H_2O_2	T275P + Heme
407	406	408	414	406
502	506	502	530	504
542 (*sh*)	545	542 (*sh*)	556	538
634	637	638	624 (*sh*)	628

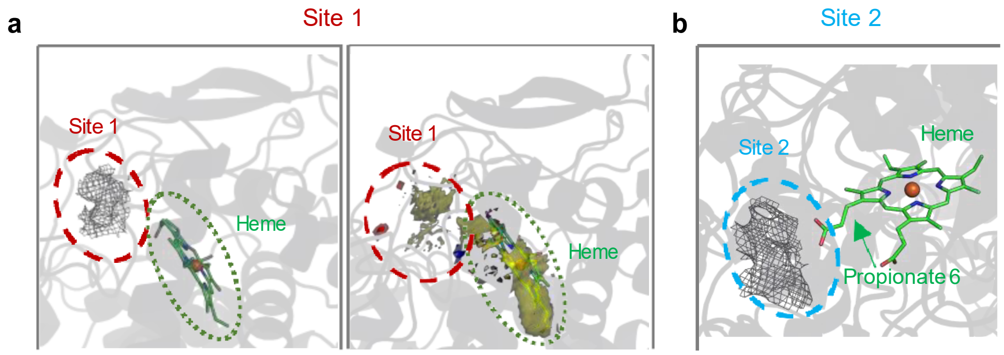

Figure S3: INH binding to KatG. a) Extra density for site 1 in protomer A near to the heme corresponding to an identified hotspot (contour14 cut off, see methods). The hot spots are shown in yellow (hydrophobic), blue (hydrogen donor) and red (hydrogen acceptor) and the extra density for INH as a grey mesh. The heme is indicated by a green dashed circle and the binding site of INH as a red dashed circle. **b)** Extra density for site 2 in protomer A identified near to propionate 6 of the heme. Site 2 is indicated with a blue dashed circle. Related to Figure 4.

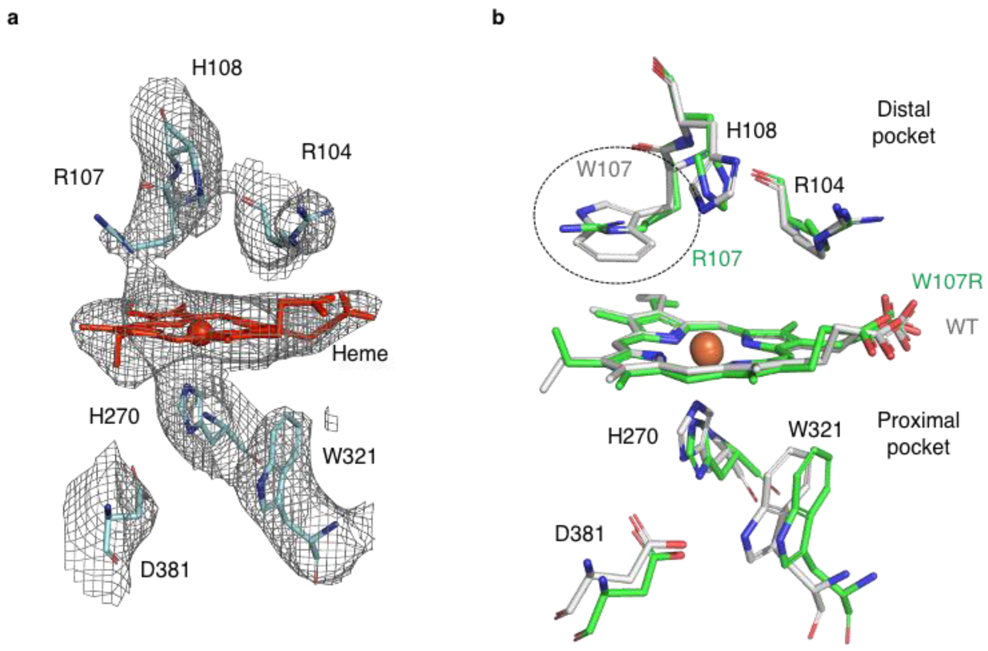

Figure S4: Active site of W107R. a) The active site of W107R, density is shown as a grey mesh, the heme in red, residues as blue sticks and labelled. **b)** Comparison between WT and W107R heme active sites. WT is shown in grey and W107R in green. The mutation in W107R is highlighted by a dashed circle. Related to Figure 5.

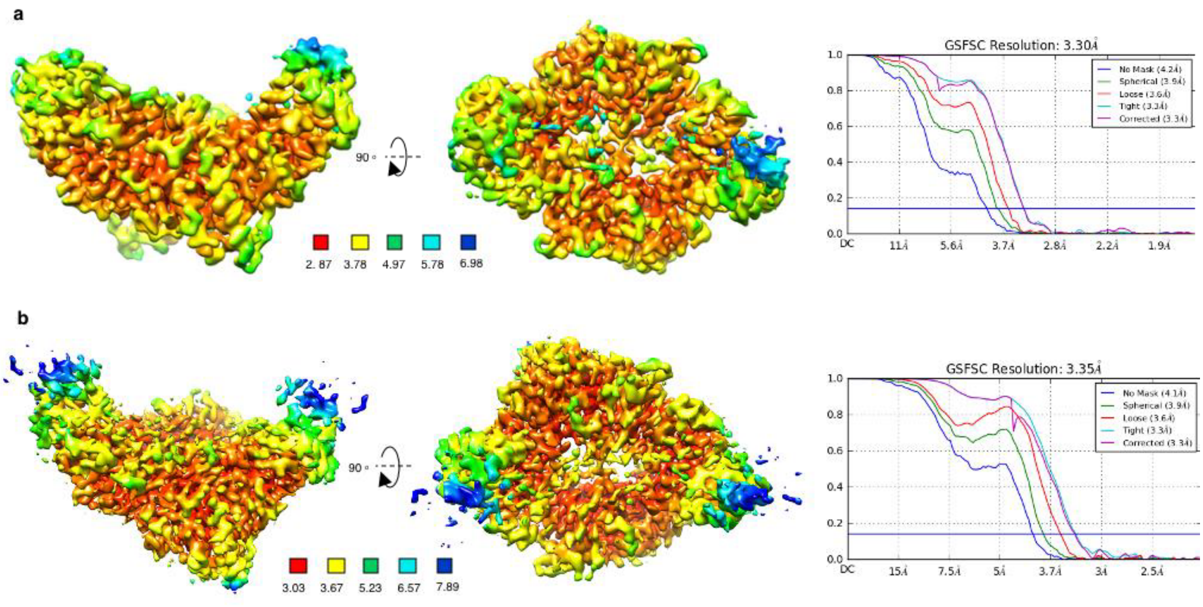

Figure S5: Local resolution maps and FSC curves of a) W107R and **b)** T275P. The coloured key below the maps indicates the resolutions and corresponding colours. Related to Figure 5.

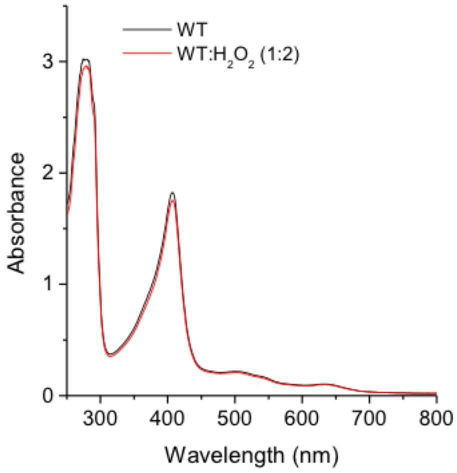

Figure S6: H_2O_2 added to WT KatG. WT KatG shown in a black line before H_2O_2 and in a red line after the addition of 2 equivalents of H_2O_2 showing no change in the UV-Visible absorbance spectrum. Related to Figure 6.

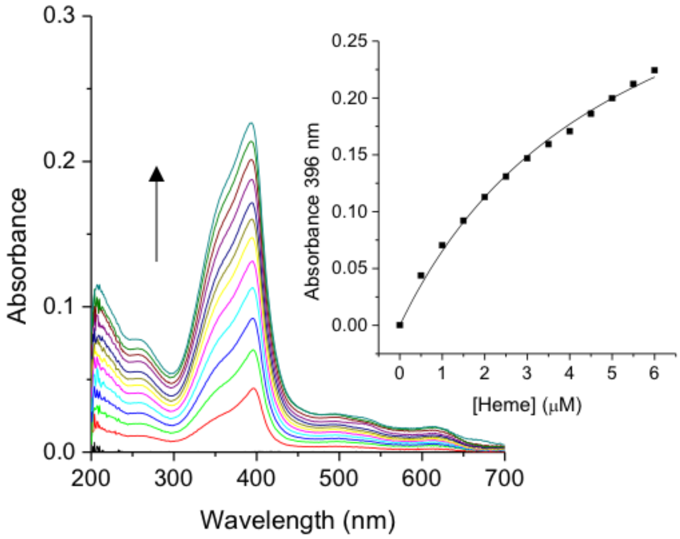

Figure S7: Addition of hemin chloride into buffer. UV-visible absorbance spectrum of hemin chloride into 20 mM NaPi, pH 7, 100 mM NaCl buffer with the arrow indicating the increase in absorbance. Inset, shows the absorbance at 396 nm plotted against heme concentration with a hyperbolic fit. Related to Figure 7.

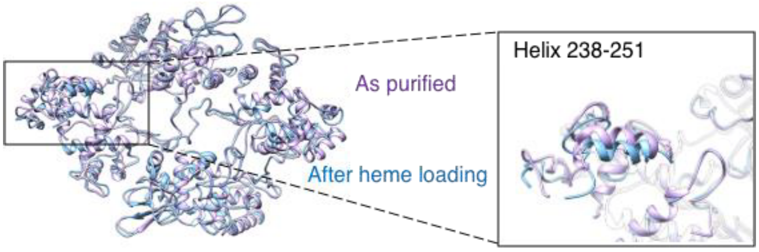

Figure S8: Comparison of the T275P KatG variant structures before and after heme loading. The structure before heme loading is shown in purple and after heme loading in blue. Inset, shows the movement of helix 238-251 between the two T275P structures. Related to Figure 5.

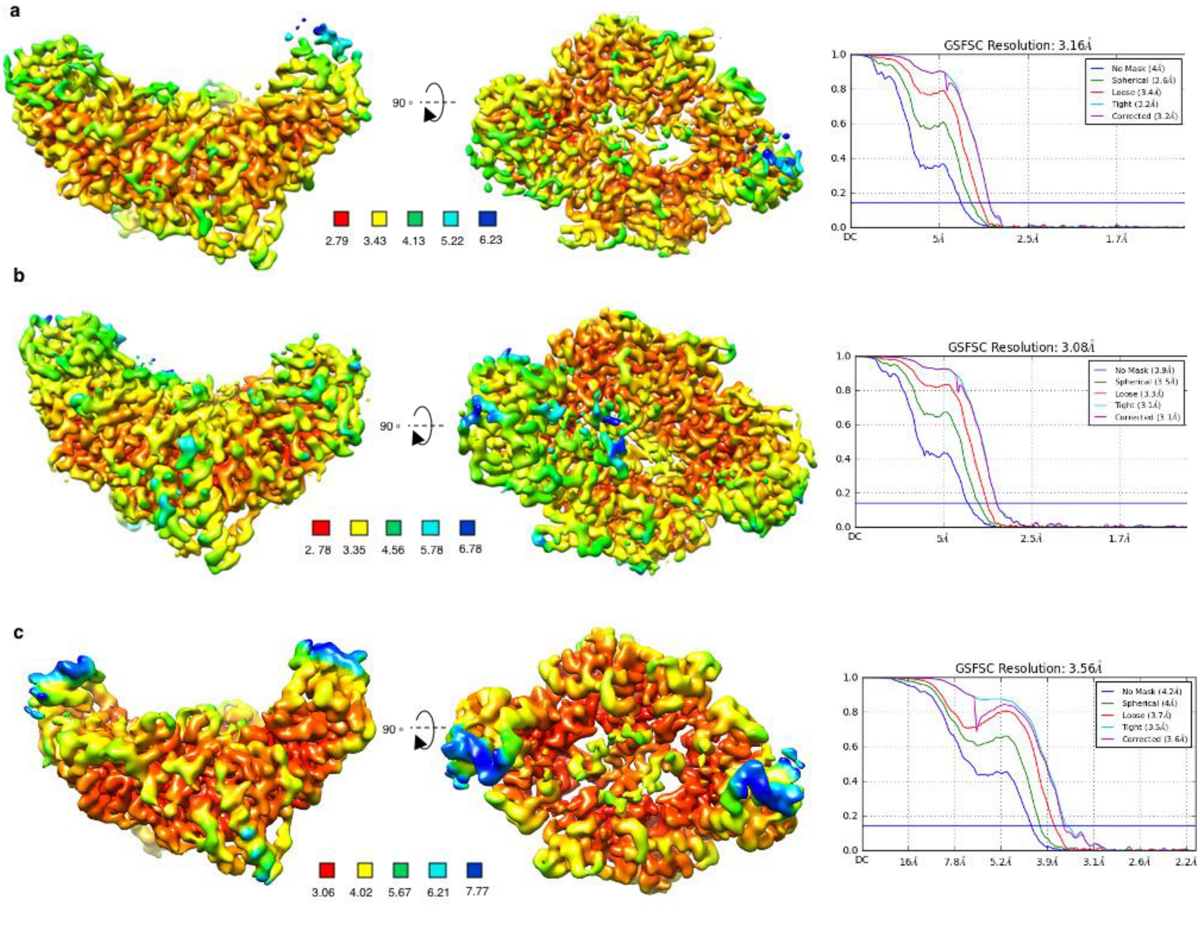

Figure S9: FSC curves and local resolution maps of W107R and T275P following heme uptake. **a)** W107R with 1 heme molecule bound, **b)** W107R with 2 heme molecules bound and **C)** T275P variant after heme uptake. Colour key below the local resolution maps indicates the resolutions. Related to Figure 8.
